# Mitral Annular Disjunction and Arrhythmic Risk: Case Series and State of the Art

**DOI:** 10.3390/biomedicines13112589

**Published:** 2025-10-23

**Authors:** Marisa Varrenti, Eleonora Bonvicini, Leandro Fabrizio Milillo, Ilaria Garofani, Lorenzo Gigli, Matteo Baroni, Alberto Preda, Marco Carbonaro, Roberto Menè, Giulia Colombo, Antonio Frontera, Raffaele Falco, Federica Giordano, Sara Vargiu, Fabrizio Guarracini, Patrizia Pedrotti, Cristina Giannattasio, Patrizio Mazzone

**Affiliations:** 1 De Gasperis Cardio Center, Niguarda Hospital, 20162 Milan, Italy; leandrofabrizio.milillo@ospedaleniguarda.it (L.F.M.); raffaele.falco@ospedaleniguarda.it (R.F.);; 2 Department of Cardiology, Santa Chiara Hospital, 38122 Trento, Italy; 3 School of Medicine and Surgery, University of Milano-Bicocca, 20126 Milan, Italy

**Keywords:** mitral, annular disjunction, arrhythmic risk, sudden cardiac death, case reports

## Abstract

**Background:** Mitral annular disjunction (MAD) is an anatomical abnormality associated with an increased risk of major arrhythmic events, regardless of the presence of mitral valve prolapse. Cardiac magnetic resonance (CMR) plays a key role in diagnosing MAD and identifying myocardial fibrosis, a marker of arrhythmic vulnerability. **Aim:** This study reports the experience of the De Gasperis Cardiology Centre at Niguarda Hospital (Milan, Italy) in managing high-risk MAD patients who underwent implantable cardioverter–defibrillator (ICD) implantation and describes their main clinical characteristics. **Methods:** Between January 2020 and April 2025, five patients with MAD who received ICDs were identified and monitored remotely. Although the small sample size limits generalizability, the objective was to characterize factors associated with arrhythmic susceptibility. **Results:** Four patients exhibited documented ventricular arrhythmias: two with non-sustained and two with sustained ventricular tachycardia. Notably, CMR did not reveal myocardial fibrosis in two symptomatic cases, suggesting that arrhythmic vulnerability may precede detectable structural abnormalities. The observed coexistence of MAD with arrhythmogenic cardiomyopathies and channelopathies underscores the relevance of comprehensive genetic evaluation in these patients. **Conclusions:** MAD should be considered a potential arrhythmogenic substrate rather than a benign anatomical variant. A multimodal diagnostic approach and individualized risk stratification—potentially integrating genetic findings—are essential for optimal patient management.

## 1. Introduction and Definition

In 1981 Bharati et al. [1] performed histological identification and description of mitral annular disjunction (MAD) as “the mitral valve anchored completely on the atrial side with an elongated annulus in a floppy mitral valve”. In 1986, Hutchins et al. [2] described a separation between the left atrioventricular junction and the left ventricular (LV) free wall in a cohort of patients using the term ‘disjunction’, which was also observed in normal hearts. In contemporary medical literature, the term “mitral annular disjunction (MAD)” is defined as a separation between the mitral valve (MV) annulus and the left ventricle (LV) myocardium. This condition is distinguished from “pseudo MAD,” a condition in which the MV leaflet is in close proximity to the atrial wall, resulting in a similar appearance without the presence of a distinct separation between the atrioventricular junction and the LV myocardium. Pseudo MAD it is most often observed in diastole, when the measured distance may appear increased despite the absence of true disjunction. Unlike MAD, pseudo-MAD is not associated with significant leaflet prolapse or curling, and it shows no correlation with late gadolinium enhancement (LGE) or myocardial fibrosis. Historically, MAD has been regarded as an uncommon occurrence. However, recent studies utilizing the analysis of cardiac magnetic resonance (CMR) or computer tomography (CT) images demonstrated a prevalence of MAD reaching up to 96% in the general population [3,4].

Mitral valve prolapse (MVP) has been identified as the most prevalent valve condition in Western countries [5]. It is characterized by the displacement of the leaflets in the left atrium during systole, with fibro myxomatous degeneration and fibroelastic deficiency being the most frequent responsible alterations. With the exception of cases of mitral regurgitation (MR) and left ventricular (LV) modifications, mitral valve prolapse (MVP) has a predominantly favourable outcome. Nonetheless, the likelihood of sudden cardiac death (SCD) in patients with mitral valve prolapse (MVP) has been estimated to be 1.75–2.3 times higher than in the general population [6]. In a recent study, the yearly incidence of severe ventricular arrhythmias (VAs) was approximately 4% in patients with MVP and no previous severe events [7,8], but only a small number of cases have been linked to poor prognosis [9].

The identification of a MVP phenotype associated with an increased risk of VAs has been proposed. This phenotype is characterized by mitral annular disjunction (MAD), which has been demonstrated to be one of the strongest arrhythmic risk factors [9].

The presence of left ventricular wall myocardial fibrosis, as documented on cardiac MRI, is another key factor in determining arrhythmic risk in patients with MVP. A study of 474 patients with MVP demonstrated that the presence of myocardial fibrosis predicts major events such as syncope, sustained ventricular tachycardia, and sudden cardiac death. In reference to the presence of MAD, Figliozzi et al. demonstrate how, with comparable documentation of MAD in the two groups with LGE+ vs. LGE−, the length of MAD was greater in patients with LGE+, hypothesizing that this feature may impact the occurrence of fibrosis and thus increased arrhythmic risk [10,11].

The correlation between MVP and MAD is unclear. The prevalence of MAD in patients with MVP varies between 16% and 32.6% [9,12,13,14,15] and the prevalence of MVP in patients with MAD is 78% [16]. Although MAD in MVP is a recognized risk factor for ventricular arrhythmias, the exact correlation between MVP, MAD and arrhythmias remains a point of discussion. Indeed, while Verhulst et al. [17] demonstrated that mitral valve abnormalities increase the arrhythmic risk in MAD patients, Dejgaard et al. [16] identified an independent correlation between MAD and more severe arrhythmias in patients with MAD but without MVP. However, it has been noted that ventricular arrhythmias increase over time in MAD patients, and mortality does not increase in the first 10 years after diagnosis, but only when VAs are detected [9].

In 2022, the European Society of Cardiology published a consensus document defining Arrhythmogenic Mitral Valve Prolapse (AMVP) as MVP (with or without MAD) combined with frequent and/or complex ventricular arrhythmia in the absence of any other well-defined arrhythmic substrate (e.g., primary cardiomyopathy, channelopathy, active ischemia, or ventricular scarring due to another defined etiology), regardless of mitral regurgitation severity.

It is currently recognized that MAD may be preceded by phenotypes not yet associated with overt arrhythmias, but which are considered ‘at risk’ of developing malignant arrhythmias [5].

This study reports the experience of the De Gasperis Cardiology Centre at Niguarda Hospital (Milan, Italy) in managing patients diagnosed with mitral annular disjunction (MAD) who were considered at high risk for arrhythmias and underwent implantable cardioverter–defibrillator (ICD) implantation. The aim was to describe the clinical and individual characteristics of this patient population. Table 1 shows the main characteristics of the clinical cases reported below.

Patients were identified through the institutional database of individuals who received ICD implantation between January 2020 and April 2025 and were subsequently followed up via the remote monitoring system of the Electrophysiology Department of our Centre.

## 2. Case Series

### 2.1. Case 1

A 50-year-old male patient with no significant comorbidities and no family history of sudden cardiac death or cardiovascular disease presented with a pre-syncopal episode during exertion in January 2023.

**Figure 1:**
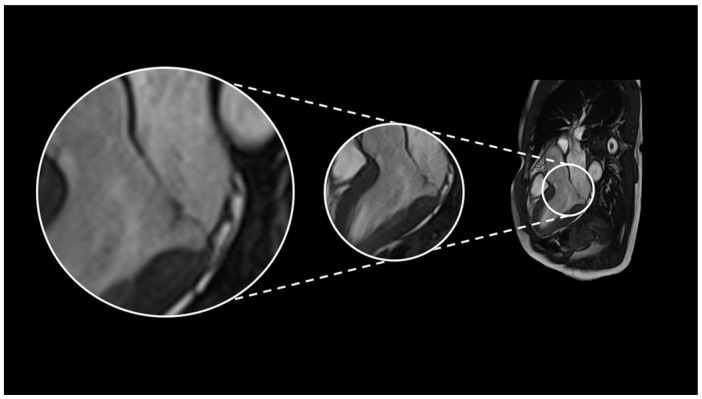
Cardiac magnetic resonance imaging (long-axis three-chamber view) demonstrates mildly redundant, prolapsing mitral-valve leaflets with a mitral annular disjunction (MAD) measuring 4 mm. Cine CMR sequences further reveal curling of the basal inferolateral and anterolateral left-ventricular walls, accompanied by mild mitral regurgitation.

**Figure 2:**
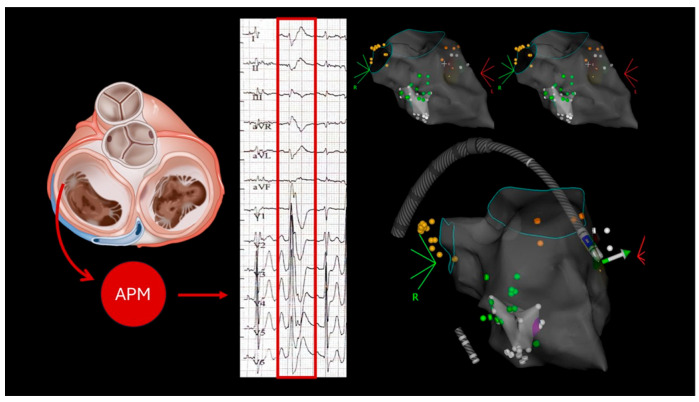
Surface ECG analysis suggested a probable origin of the clinical ventricular ectopy from the anterolateral papillary muscle (ALPM). A combined retrograde transaortic and transseptal approach was used to access the left ventricle. During electroanatomical mapping, mechanically induced premature ventricular contractions (PVCs), showing a morphology consistent with the clinical pattern (positive concordant right bundle branch block and intermediate axis), were observed, confirming the ALPM as the site of origin. Radiofrequency catheter ablation (RFCA) was successfully performed at this location via the retrograde aortic route, resulting in resolution of the PVCs.

A 24 h Holter ECG revealed rare single monomorphic ventricular extrasystoles and three runs of non-sustained ventricular tachycardia (NSVT) lasting up to four beats. A transthoracic echocardiogram was performed, which revealed mild to moderate mitral regurgitation, with biventricular systolic function found to be at the lower limits of normal. He underwent an exercise test, which was interrupted at 83% of the maximum theoretical frequency due to the onset of asymptomatic ventricular bigeminy, which regressed spontaneously during recovery. Cardiac magnetic resonance imaging (MRI) showed mitral valve prolapse, with 4 mm MAD and preserved biventricular systolic function (Figure 1). No evidence of enhancement, edema or areas of fat replacement was observed. The patient was initiated on beta-blocker therapy (bisoprolol 1.25 mg/day), which effectively controlled symptoms until May 2024, when the patient experienced recurrent exertional pre-syncope without angina or palpitations. A physical examination was conducted, which revealed adequate hemodynamic compensation. However, electrocardiographic (ECG) analysis revealed sinus bradycardia, with indications of early left ventricular overload and QRS fragmentation in leads D3 and aVF. As recorded in the 24 h Holter monitoring results, two episodes of rapid monomorphic ventricular tachycardia were documented, with each episode lasting approximately 25 s. These episodes were found to correlate with symptomatic presyncope. The patient was subsequently admitted for coronary angiography, a procedure which ruled out the presence of coronary artery disease. Echocardiography revealed that the biventricular function had been preserved, and this finding confirmed the presence of mild-to-moderate mitral regurgitation, which was due to anterior mitral valve prolapse. Due to the considerable ventricular arrhythmic burden, the patient underwent catheter ablation targeting the arrhythmic focus originating from the anterolateral papillary muscle (Figure 2), followed by implantation of an extracardiac implantable cardioverter–defibrillator (ICD). There were no defibrillator interventions and no significant arrhythmic recurrences in the annual follow-up.

### 2.2. Case 2

The second case concerns a 58-year-old male patient. The patient’s medical history included systemic arterial hypertension, type II diabetes mellitus, a body mass index in the overweight category, a previous deep vein thrombosis of the right upper limb with MTHFR mutation, and hyperhomocysteinemia. In the absence of prodromal symptoms, a syncopal episode was experienced, subsequently accompanied by palpitations and dizziness. The patient presented to the emergency department. The electrocardiogram (ECG) revealed symptomatic non-sustained ventricular tachycardia (NSVT) (heart rate (HR) 300 beats per minute (bpm), duration 8 s) with inverted T waves in leads DIII and aVF. A detailed echocardiographic examination revealed mild mitral valve prolapse, with the potential for MAD. Following the attainment of arrhythmic stability, a coronary angiogram was conducted, which revealed the absence of coronary artery disease. Cardiac magnetic resonance imaging revealed normal biventricular size and function, confirming MAD measuring 5 mm in the inferior region without associated prolapse. The evaluation for myocardial edema, fatty infiltration, and fibrosis yielded negative results. Consequently, a defibrillator was implanted, and beta-blocker therapy was initiated.

### 2.3. Case 3

The third case concerns a 43-year-old female patient with a medical history of essential thrombocythemia, who was treated with acetylsalicylic acid. A recent cardiological evaluation revealed frequent ventricular extrasystoles (11,000 in 24 h), primarily isolated but occasionally occurring in pairs or triplets. Echocardiography revealed a left ventricle that was above the upper limits of normal size, indicating left ventricular dysfunction due to diffuse hypokinesia. Additionally, mild mitral regurgitation was observed, attributed to prolapse. In contrast, the right heart chambers were found to be within normal limits. The therapeutic approach was initiated with a dosage of metoprolol 50 mg administered twice daily. A cardiac MRI was performed, which documented mild LV dilatation (DTD 61 mm, VTD 94 mL/m^2^) with preserved wall thickness, except for the mid-apical lateral wall, which appeared dyskinetic and thinned. Septal dyssynchrony was also noted, with an ejection fraction of 41%. No late gadolinium enhancement (LGE) indicative of myocardial fibrosis was detected. STIR sequences were negative, ruling out the presence of myocardial edema, and both T1 and T2 mapping values were within normal limits, suggesting the absence of active inflammation. A mild pericardial effusion was also observed. Furthermore, the right ventricle exhibited mild dysfunction, characterized by an ejection fraction of 44%. The findings, when considered collectively, suggest a picture consistent with dilated cardiomyopathy.

A 24 h Holter ECG was conducted, which confirmed a high ventricular arrhythmic burden. This included 19,759 polymorphic ventricular ectopic beats (18.5%), comprising 715 couplets and 6 triplets. The baseline ECG exhibited sinus rhythm at a rate of 61 beats per minute (bpm), with a PQ interval of 178 milliseconds (ms). The QRS voltages in all peripheral leads were low, with fragmentation observed in leads D3, aVL, and aVF (in the inferior-lateral region). In contrast, the voltages in the precordial leads remained stable, and the QRS duration fell within the normal range. The presence of negative T waves in the inferior leads, in conjunction with biventricular bigeminy exhibiting right bundle branch block morphology, was duly noted. The medical therapy was intensified with the addition of eplerenone and dapagliflozin.

The patient was admitted for further diagnostic procedures. A subsequent cardiac MRI revealed a mild reduction in biventricular systolic function, mild mitral regurgitation due to valve prolapse, and mitral annular disjunction (MAD) measuring 7 mm. Late gadolinium enhancement (LGE) was observed at the apex of the inferolateral papillary muscle.

Due to the ventricular arrhythmic profile exhibited by the patient, who was diagnosed with cardiomyopathy in conjunction with mitral valve prolapse and MAD, in addition to the presence of left ventricular free wall (LGE) involving the inferolateral papillary muscle, the decision was to proceed with the implantation of an implantable cardioverter–defibrillator (ICD) as a primary prevention measure.

### 2.4. Case 4

The fourth case refers to a 34-year-old female patient who was referred following the electrocardiographic detection of a type 1 Brugada pattern during a period of hospitalization for fever and chest pain. The echocardiogram revealed a normal left ventricle size, kinesis, and biventricular systolic function, accompanied by mild atrioventricular valve insufficiency. The ECG findings were used to inform genetic testing, which revealed the p.51573G variant in the SCN5A gene. This variant was also identified in both the father and son. An endocardial electrophysiological study (EPS) was conducted, which revealed ventricular fibrillation (drive 500 ms, double extrastimulus at 220 ms). The ventricular fibrillation was effectively cardioverted with a single DC shock at 200 J.

Subsequent cardiac magnetic resonance imaging revealed normal cavity dimensions and preserved global biventricular systolic function. No evident areas of myocardial edema or adipose infiltration were detected. The mitral valve leaflets appeared slightly redundant and prolapsed, with a 6 mm MAD, accompanied by curling of the basal segments of the inferolateral and anterolateral walls of the left ventricle, and mild mitral regurgitation. Subsequent to the administration of contrast media, subsequent imaging revealed focal enhancement lesions in the posteromedial papillary muscle. Furthermore, tricuspid annular disjunction (TAD) measuring 4 mm was observed.

**Figure 3:**
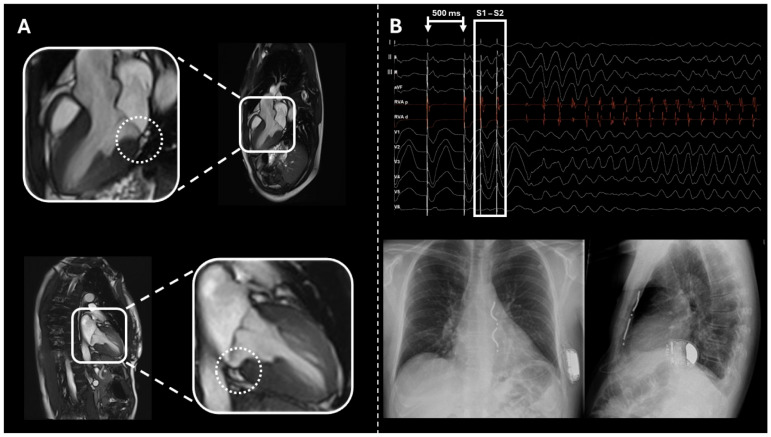
(**A**) Cardiac magnetic resonance imaging in the three-chamber view (upper panel) and two-chamber view (lower panel) demonstrating a mitral annular disjunction (MAD) measuring 6 mm, evident as a separation between the posterior mitral annulus and the basal inferolateral left ventricular myocardium during systole. (**B**) Upper section: Intracardiac electrophysiological study performed using a drive train of 8 stimuli at a cycle length of 500 ms, followed by 2 ventricular extrastimuli (S1–S2) at a coupling interval of 220 ms, resulting in the induction of ventricular fibrillation (VF). The arrhythmia was promptly terminated with a single 200 J external biphasic defibrillation shock, restoring sinus rhythm. Lower section: Anteroposterior and lateral chest radiographic views following implantation of an extravascular implantable cardioverter–defibrillator (EV-ICD), demonstrating appropriate lead and device placement.

In view of the results of the electrocardiogram (ECG) and the patient’s clinical history, an extravascular implantable cardioverter–defibrillator (ICD) was implanted (see Figure 3).

### 2.5. Case 5

The final case involves a 70-year-old male patient with a history of mitral valve prolapse, who was referred to our centre following the completion of a 24 h Holter ECG that documented sustained ventricular tachycardia lasting 42 beats. The diagnosis of coronary artery disease was excluded by coronary CT. Cardiac magnetic resonance imaging revealed mitral valve prolapse associated with an 8 mm MAD and intramyocardial late gadolinium enhancement in the mid-basal septum. The patient was then admitted for defibrillator implantation in prevention of sudden cardiac death.

## 3. Discussion

We selected five cases of patients with mitral annular disjunction (MAD) who underwent ICD implantation. This could be considered a limitation of the study; however, our aim was to describe cases with a high arrhythmic risk in order to analyze their characteristics and identify factors contributing to increased arrhythmic vulnerability. 

The presence of MAD—whether or not associated with mitral valve prolapse, myocardial fibrosis, or electrocardiographic abnormalities—was found to significantly influence arrhythmic risk and, consequently, the decision regarding protection from sudden cardiac death.

The documentation of ventricular arrhythmias is a recognized risk factor for major arrhythmic events and supports the identification of patients who may benefit from preventive strategies against sudden cardiac death. In our case series, four out of five patients had documented arrhythmias: two with non-sustained ventricular tachycardia and two with sustained ventricular tachycardia.

Baseline ECG abnormalities also appear to be useful for arrhythmic risk assessment in patients with MAD. In two of the five cases, the patients demonstrated negative T waves in the inferior leads. Although not always correlated with fibrosis, these findings may help identify patients requiring closer clinical follow-up.

Cardiac magnetic resonance imaging (CMR) plays a key role in detecting myocardial fibrosis, which is associated with increased arrhythmic risk. However, no fibrosis was observed in the first two cases despite the presence of syncope-inducing symptoms. This suggests that arrhythmic risk may precede the phenotypic expression of myocardial stress, as evidenced by fibrosis.

Moreover, the correlation between MAD and cardiomyopathies with an arrhythmic phenotype—as well as channelopathies, exemplified by the case of a young female patient without fibrosis—highlights the need for comprehensive genetic evaluation in this population. Current knowledge in this field remains limited, emphasizing the importance of further research.

### 3.1. Mechanisms of Arrhythmias

The presence of MAD has been shown to result in excessive mobility of the mitral annulus, which in turn leads to an abnormal systolic curling of the myocardium in the posterior mitral ring [18]. This process is likely to cause isolated and asymmetrical left ventricular (LV) hypertrophy, which is localized in the basal inferolateral segment [19]. The phenomenon of hypertrophy of the papillary muscles (PMs) has also been documented, a likely consequence of altered mechanical load and forces acting upon them [19]. Increased wall stress in this region has been observed to result in repetitive microtrauma and subsequent fibrotic replacement, as evidenced by histological studies and LGE on CMR imaging in vivo subjects [20]. This fibrotic substrate has been shown to be proarrhythmic [21], and the associated mechanical stretch of the mitral annulus may facilitate the development of ventricular arrhythmias. Fibrosis has been shown to induce repolarisation abnormalities, while mechanical stretch has been demonstrated to shorten the action potential duration and lower the resting diastolic potential. This, in turn, has been shown to promote early after-depolarisations and triggered activity [22]. Mechanical stretching of cardiac cells activates stretch-activated channels (SACs), which are voltage-sensitive ion channels permeable to cations such as Na^+^, Ca^2+^, and K^+^. Activation of SACs can depolarize the cell membrane, increasing cellular excitability. This depolarization can lead to a shortening of the action potential duration (APD) and a reduction in the diastolic resting potential. These electrophysiological changes enhance the likelihood of abnormal electrical activity, thereby promoting arrhythmogenesis [23]. It is important to consider that not all MAD cases are associated with fibrosis [9], suggesting that the development of tissue alterations and consequently the arrhythmic risk may be progressive.

### 3.2. Arrhythmic Risk Stratification

In patients diagnosed with MVP, the stratification of arrhythmic risk has been a subject of extensive debates, with the aim of identifying patients who are at elevated risk of SCD. While it is widely accepted that MAD is a significant arrhythmic risk factor in patients with MVP, the precise stratification of arrhythmic risk in MAD patients with or without MVP remains a subject of ongoing research.

The existing literature suggests that younger age is associated with an increased risk of arrhythmia in MAD [16], the role of female sex as a predictor factor remains a subject of debate [20,24]. Furthermore, presyncope and unexplained syncope have been identified as indicators of patients with a higher arrhythmic risk in comparison to isolated palpitations and asymptomatic status [16,25].

Electrocardiography and ambulatory ECG monitoring are recognized as essential tools for arrhythmic risk stratification. Abnormal repolarisation, consequent to unbalance forces that act upon the myocardium and PMs, is revealed on the surface ECG through T wave inversions (TWI) or biphasic T waves, predominantly in the inferior and lateral leads. These have been demonstrated to be independently associated with malignant arrhythmias [6,9]. In our case series, two patients demonstrated inferior T-wave inversions on baseline ECG. In one patient, cardiac MRI additionally revealed evidence of myocardial fibrosis, whereas the other showed no such findings. Both patients had documented TVNS on ECG monitoring. Of note, one of these patients was symptomatic with syncope.

However, it should be noted that not all reports concur with this assertion, and the existence of a direct association with increased mortality remains a subject of debate [20]. Finally, Kaya et al. [26] proposed that fragmented QRS could predict VAs in MVP patients, although further data are needed to validate this hypothesis. Premature ventricular contractions (PVCs) and non-sustained ventricular tachycardia (NSVT) originating from the PMs, the mitral annulus and the basal portion of the left ventricle (LV) are frequently observed. Although a history of NSVT is associated with increased arrhythmic risk, the question of whether the burden of PVCs has the same prognostic value remains the subject of debate [16,27].

Multimodality imaging, incorporating echocardiography and CMR, is of crucial importance in the diagnosis and risk stratification of this disease. In the domain of 2D echocardiography, MAD is typically identified and quantitated in the parasternal long-axis view during the late systolic phase of the cardiac cycle. CMR is also useful for a more detailed characterization of the extension and localization of the MAD and overall for the differential diagnosis with pseudo-MAD. It is imperative that both imaging techniques are employed in order to assess the dimensions and function of the left ventricle (LV), the size of the left atrium (LA), the morphology of the mitral valve, and the degree of mitral regurgitation. The accurate evaluation of the extension and localization of MAD has been demonstrated to have a major impact on arrhythmic risk stratification. In two cohorts of idiopathic ventricular fibrillation (IVF) patients [17,28], inferolateral MAD was observed to be significantly more prevalent than other localizations. In such cases, the length of the MAD did not demonstrate a correlation with the risk of arrhythmia. However, Carmo et al. [29] found that a MAD length greater than 8.5 mm was able to predict NSVT. The present findings are consistent with those reported in other studies [3,15]. Associations between MAD length, myocardial fibrosis, and cardiac death have also been described [19].

Myocardial fibrosis, secondary to mechanical stress, has been proposed as a predictor of arrhythmic risk. LGE on CMR, an indirect marker of fibrosis, has typically been identified as patchy areas in the infero-basal LV and mid-wall of the PMs [15]. Perazzolo Marra et al. [19] reported that LGE is more frequently present in patients with MAD than in those with isolated MVP, and that it increases with the length of MAD [10]. Several studies have confirmed the association between LGE and ventricular arrhythmias in MVP patients [10,11,30], although data on isolated MAD remain limited [9,16,31]. However, research has demonstrated that arrhythmia focuses in MAD are known to originate from fibrotic areas [19,20]. The hypothesis that T1 mapping may have a role in arrhythmic stratification in MVP has been advanced [30]. Moreover, recent data suggest a potential link between basal inferolateral hypertrophy and an elevated risk of SCD [32].

In addition to myocardial modifications, the presence of MAD and MVP has been linked to electromechanical dyssynchrony. The calculation of left ventricular mechanical dispersion (MD) through strain analysis has been utilized in both ischemic and non-ischemic cardiomyopathies to quantify electromechanical dyssynchrony, and has been demonstrated to correlate with arrhythmic risk [33]. Increased MD has also been found in MVP with increased arrhythmic risk [34], but specific studies for MAD has not been conducted yet. Similarly, post-systolic shortening has been associated with arrhythmogenesis in MVP, though evidence specific to MAD is limited [35].

Although MAD and MVP are likely distinct pathophysiological entities, the two diseases are diagnosed together and MAD has been recognized as the most important predictor of arrhythmic events in MVP. Consequently, when MAD co-exists with MVP, classical MVP-related risk markers must also be taken into account [6,9]. These risk markers include, but are not limited to, bileaflet prolapse, myxomatous degeneration, mitral regurgitation, and LA or LV dilation.

Dejgaard et al. [16] observed that MVP patients with MAD may have less severe arrhythmias, potentially due to earlier diagnosis and treatment compared to isolated MAD cases.

**Figure 4:**
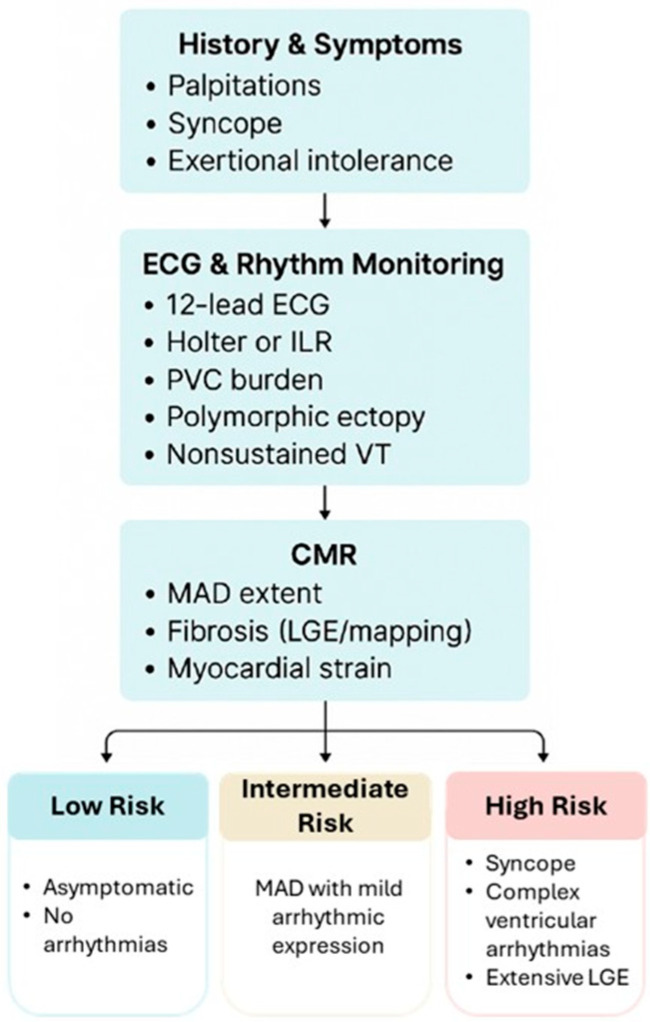
Possible flow chart for arrhythmic risk stratification in patients with MAD.

It has been hypothesized that a genetic predisposition may underlie non-syndromic MVP [36,37]. However, the correlation between MAD and genetically determined arrhythmic predisposition remains hypothetical [2]. Furthermore, the assessment of biomarkers has not yet yielded results in this regard. Given the heterogeneity of MAD manifestations—ranging from incidental findings without fibrosis or ventricular arrhythmias to overlapping channelopathies or cardiomyopathies—a pragmatic, multimodal risk assessment is warranted. Clinicians should begin with a detailed history of symptoms, focusing on palpitations, syncope, or exercise intolerance. This should be complemented by a 12-lead ECG and an extended Holter monitor or implantable loop recorder to evaluate the burden of PVCs, polymorphic ectopy, or nonsustained ventricular tachycardia. Echocardiography remains essential for assessing MVP, leaflet redundancy, and the presence of MAD, while cardiac magnetic resonance imaging (CMR) adds further value by quantifying the extent of MAD and identifying both the presence and degree of fibrosis. A family history of sudden cardiac death or inherited arrhythmic syndromes should prompt heightened vigilance. Taken together, these data points can be integrated into a practical risk profile (see Figure 4):

- Low risk: asymptomatic, short MAD segments, no arrhythmia.
- Intermediate risk: MAD with mild arrhythmic expression.
- High risk: syncope, complex ventricular arrhythmias, or extensive LGE.

Further research is needed on this topic, particularly to clarify the genetic aspects in this group of patients.

### 3.3. Therapeutic Considerations

The current literature on arrhythmia management, especially in patients with MVP, is limited, and information regarding patients with MAD is extremely limited. While pharmacological therapy has been shown to alleviate symptoms and improve quality of life, there is no evidence to suggest that it has an impact on mortality [27]. Of all the anti-arrhythmic drugs, flecainide has produced encouraging results [7]. Current ESC guidelines for ventricular arrhythmias recommend ICD implantation for primary and secondary prevention [38], have suggested that ICD implantation should be considered for MVP patients presenting with unexplained syncope and non-sustained ventricular tachycardia (NSVT) likely arising from the mitral apparatus, or for patients presenting with unexplained syncope or NSVT and exhibiting two or more phenotypic risk features (e.g., T wave inversion in the inferior leads, repetitive polymorphic premature ventricular complexes (PVCs), MAD, redundant mitral valve (MV) leaflets, an enlarged left atrium, an ejection fraction of ≤50%, or late gadolinium enhancement (LGE) within the mitral apparatus. Catheter ablation is also performed according to general guidelines for ventricular arrhythmias [38]. Specific data on VA ablation in MAD are currently lacking. However, a case series involving 14 patients with mitral valve prolapse (MVP) who underwent catheter ablation reported a procedural success rate of 98% [39]. Surgical correction of severe mitral regurgitation (MR) has been shown to restore life expectancy in patients without VAs and reduce VAs in patients with MVP [9,24]. However, it is unclear whether this correction alone is sufficient to eliminate the need for ICD implantation in patients with arrhythmia.

## 4. Conclusions

It is evident that prospective studies incorporating larger case series are imperative to further delineate the arrhythmic risk in patients with MAD, with or without concomitant mitral prolapse, with the objective of identifying those who are predisposed to major arrhythmic events.

In conclusion, MAD should be regarded not only as an anatomic variant but also as a potential arrhythmogenic heart disease that necessitates multimodal evaluation and individualized risk assessment. A more comprehensive understanding of its pathophysiology and correlation with possible genetic variants is imperative to enhance patient outcomes through early diagnosis and targeted therapies.

## Figures and Tables

**Table 1:** Main Characteristics of Patients with Mitral Annular Disjunction (MAD) and High Arrhythmic Risk.

Age	Gender	Symptoms	ECG	Arrhythmias	CMR	MVP	Mitral Regurgitation	LVEF	Therapy	EPS
50	M	Pre-Syncope after exercise		SVT	- 4 mm MAD - No fibrosis	Present	MODERATE	55%	Bisoprolol	Performed, negative
58	M	Syncope	Negative T waves in inferior leads	- NSVT	- 5 mm MAD - No fibrosis	Present	MILD	55%	No	Not performed
43	F	-	- Low peripheral voltages - Negative T waves in inferior leads	- NSVT	- 7 mm MAD - Infero-lateral papillary muscle LGE	Present	MILD	44%	Bisoprolol	Not performed
34	F	-	Spontaneous type 1 Brugada pattern	- None	- 6 mm MAD - 4 mm TAD - Posterior medial papillary muscle LGE	Present	MILD	55%	No	Performed, positive for VF
70	M	-		- SVT	- 8 mm MAD - Septal LGE	Present	MILD	50%	No	Performed, Negative

CMR = Cardiac Magnetic Resonance, SVT = Sustained Ventricular Tachycardia, NSVT = Non-Sustained Ventricular Tachycardia, VF = Ventricular Fibrillation, MAD = Mitral Annular Disjunction, TAD = Tricuspid Annular Disjunction, LGE = Late Gadolinium Enhancement, EPS = Electrophysiological Study, MVP = Mitral Valve Prolapse, LVEF = Left Ventricular Ejection Fraction.

## Data Availability

Data are contained within the article.

## Abbreviations

CMR	Cardiac Magnetic Resonance
EPS	Electrophysiological Study
LGE	Late Gadolinium Enhancement
MAD	Mitral Annular Disjunction
MVP	Mitral valve prolapse
NSVT	Non-Sustained Ventricular Tachycardia
SVT	Sustained Ventricular Tachycardia
VF	Ventricular Fibrillation
TAD	Tricuspid Annular Disjunction
